# Effects of plasma potassium on myocardial function: a POTCAST substudy

**DOI:** 10.1186/s44348-026-00068-7

**Published:** 2026-04-02

**Authors:** Tharsika Sakthivel, Ulrik CG Winsløw, Chaoqun Zheng, Elisabeth Margrethe Danielsen, Helle S Bosselmann, Michael Vinther, Niels E Bruun, Henning Bundgaard, Christian Jons, Niels Risum

**Affiliations:** 1 Department of Cardiology, Heart Centre, Copenhagen University Hospital, Copenhagen, Denmark; 2 Department of Cardiology, Zealand University Hospital, Roskilde, Denmark

**Keywords:** Potassium, Global longitudinal strain, Myocardial Contraction, Echocardiography, Deformation imaging

## Abstract

**Background:**

Small studies suggest that variations in plasma potassium (p-K) levels may affect cardiac contractile function. A substudy to the recently published POTCAST trial demonstrated short-term improvements in myocardial function in patients randomized to potassium-increasing treatment. However, the long-term effects of increasing p-K on cardiac function remain unclear. This study aimed to investigate whether treatment that increases p-K improves diastolic and systolic myocardial function as assessed by echocardiography during long-term follow-up in a Danish implantable cardioverter-defibrillator (ICD) cohort.

**Methods:**

The POTCAST trial randomized patients with an ICD (1:1) to either usual therapy (control group) or treatment with oral potassium supplements and/or mineralocorticoid receptor antagonists (high-normal potassium group). In this substudy, consecutive patients from both arms of the POTCAST trial were included. Echocardiography was performed at baseline and repeated after > 6 months for the current study to compare changes in left ventricular ejection fraction (LVEF), global longitudinal strain (GLS), global constructive work (GCW), and diastolic parameters (E, e’, and E/e’) between the high-normal potassium and control groups.

**Results:**

A total of 289 patients (mean age, 58 ± 13.4 years; 77.5% male) were included in the study. The median follow-up time between baseline and follow-up echocardiography was 729 days (interquartile range, 511–986 days). From baseline to follow-up the high-normal potassium group had an increase in mean difference in p-K of 0.22 mmol/L (95% confidence interval [CI], –0.31 to –0.13; P < 0.001) compared to the control group. In the high-normal potassium group e’lat increased by 0.77 cm/sec (95% CI, 0.12–1.40; P = 0.020), and E/e’lat decreased by –1.15 (95% CI, –2.1 to –0.25; P = 0.013) on average compared to the control group. No significant differences were observed in changes in other diastolic parameters. For systolic function, no significant differences were found between groups in terms of change in LVEF (–0.6%; 95% CI, –2.17 to 1.02; P = 0.475), GLS (–0.26%; 95% CI, –0.8 to 0.4; P = 0.472), or GCW (–3.99 mmHg; 95% CI, –89.5 to 81.6; P = 0.927).

**Conclusions:**

In contrast to previous short-term findings, when myocardial function was assessed by echocardiography, long-term potassium-increasing treatment led to only minor improvements in diastolic function in a contemporary Danish ICD cohort.

**Trial Registration:**

ClinicalTrials.gov identifier: NCT03833089.

**Supplementary Information:**

The online version contains supplementary material available at 10.1186/s44348-026-00068-7.

## Background

The influence of plasma potassium (p-K) on the electrophysiology of cardiomyocytes is well established [1–3]. Potassium is the primary cation involved in the repolarization phase of the action potential and plays an important role in stabilizing membrane potential [4, 5]. Consequently, derangements in p-K levels can increase the risk of malignant arrhythmias [6, 7] and mortality [4, 8]. Systolic and diastolic phases of the cardiac cycle occur as a result of the propagation of action potentials through the myocardium [9]. Therefore, it is reasonable to assume that the electrophysiological properties of p-K may impact myocardial mechanical function. However, further studies are needed to investigate this association.

In patients receiving heart failure treatment, mineralocorticoid receptor antagonists (MRAs) reduce the risk of hospitalization for worsening heart failure and all-cause mortality [10–12]. This beneficial effect might be in part mediated by MRA-induced increases in p-K levels, but it has never been thoroughly investigated. Additionally, a study in dogs demonstrated that potassium depletion impaired both systolic and diastolic function [13], and these findings aligned with studies in patients with chronically low p-K caused by primary hyperaldosteronism [14, 15]. Thus, high as well as low p-K levels may affect the mechanical function of the myocardium.

We have recently identified an association between potassium-increasing treatment and improvements in global longitudinal strain (GLS) and diastolic parameters, including early diastolic mitral annular velocity (e’) and E/e’ ratio, as assessed by echocardiography [16]. However, the study involved only 6 weeks of treatment prior to follow-up echocardiography. In the current study, we aimed to investigate the long-term effects (> 6 months) of an intervention to increase and stabilize p-K at high-normal levels on myocardial function.

## Methods

### Ethics statement

The present study is a substudy of the POTCAST trial. The study was approved by the Regional Danish Committee on Health Research Ethics (Regional Videnskabsetisk Komité) (approval no. H-18044908) and the Danish Data Protection Agency (approval no. VD-2018–453). Written informed consent was obtained from all patients. The study was conducted in accordance with the Declaration of Helsinki.

### POTCAST study

Patients in the current substudy were selected from the POTCAST trial (ClinicalTrials.gov identifier: NCT03833089). The study design was published previously [17, 18]. In brief, the POTCAST trial was a randomized clinical trial enrolling patients at high risk of malignant arrythmias, defined as those receiving an implantable cardioverter-defibrillator (ICD) for primary or secondary prevention. The primary objectives of the POTCAST trial were to investigate whether p-K-increasing treatment (MRA and potassium supplements) could increase and stabilize p-K levels in the high-normal range (4.5–5.0 mmol/L), and to determine whether maintaining high-normal p-K levels reduces the incidence of malignant cardiac arrhythmias and all-cause mortality.

The trial was a multicenter study enrolling 1,200 patients from Copenhagen University Hospital, Rigshospitalet; Copenhagen University Hospital, Herlev-Gentofte; and Zealand University Hospital, Roskilde. Patients were randomized 1:1 to either the control or high-normal potassium group. Baseline treatment with MRA and potassium supplements as part of usual therapy was allowed in both groups. Treatment was titrated gradually in the high-normal potassium group. MRA-naive patients started with 25 mg of eplerenone or spironolactone while those already on MRA or potassium supplements, or with a history of side effects, had individualized treatment adjustments. Every 2 weeks, treatment was doubled, and p-K levels were monitored. Once the maximum daily MRA dose (100 mg spironolactone or 50 mg eplerenone) was reached, potassium supplements were introduced and gradually increased (maximum, 4,500 mg [approximately 60 mmol] potassium chloride). This treatment plan continued until one of the following was achieved: (1) the highest tolerable dose without side effects; (2) a p-K level of ≥ 4.5 mmol/L; or (3) the maximum study medication dose. The control groups continued with their usual therapy alone. If this therapy included MRA and potassium supplements at study inclusion, no further protocol-driven adjustments were made.

### Study population

The inclusion and exclusion criteria for this substudy were similar to the POTCAST trial. Patients included were ≥ 18 years and at high risk of malignant arrhythmias, as indicated by the presence of an ICD implanted according to clinical guideline-recommended management for their cardiac diagnosis. Exclusion criteria were baseline p-K > 4.3 mmol/L, estimated glomerular filtration rate ≤ 30 mL/min/1.73 m^2^ and the inability to understand and sign informed consent. Patients were ineligible for inclusion in this substudy if they had no baseline echocardiography, if baseline echocardiography was of insufficient quality for the primary analysis, or if the echocardiography from patients in the high-normal potassium group was performed after initiation of study medication. Consecutive patients from both the high-normal potassium and the control group at the Rigshospitalet study site were invited to participate in the substudy and undergo follow-up echocardiography. Follow-up echocardiography was performed at least 6 months after inclusion in the study.

### Echocardiography

The ultrasound system used in this study was from GE Healthcare, and all echocardiographic analyses were conducted using EchoPAC ver. 206 (GE Healthcare). The primary investigator (TS) conducted all analyses. Gray-scale imaging was used to capture cine loops from three standard apical views: four-chamber, two-chamber, and apical long-axis views. Biplane Simpson method was used to obtain left ventricular ejection fraction (LVEF). Diastolic function and dysfunction were assessed according to the 2016 guidelines from the American Society of Echocardiography and European Association of Cardiovascular Imaging [19]. Measurements obtained to describe diastole were early diastolic mitral inflow velocity (E), late diastolic mitral inflow velocity (A), E/A ratio, the average of the septal (e’sept) and lateral (e’lat) early diastolic mitral annular velocities (e’), E/e’ ratio, tricuspid regurgitation gradient, and left atrial volume index. In patients with atrial fibrillation, diastolic function assessment was based on a combination of visual evaluation and the above-mentioned guideline-based flowcharts. Images for GLS analysis were acquired at a frame rate of 50 to 90 frames per second. The inner endocardial border was traced on 2D images at the end-systolic frame from three apical views (two-, three, and four-chamber views). Subsequently, speckles were tracked frame by-frame throughout the LV wall and regions of interest were defined for the basal, midventricular, and apical LV areas. Manual adjustments were performed by the operator if any segments failed to track automatically. Segments that subsequently failed to track were eventually excluded. If more than two segments failed to track in more than one projection, GLS measurement was considered unattainable for that patient. The mean strain of all successfully tracked segments was used to compute GLS. GLS was calculated as the mean peak negative longitudinal strain during systole, following the framework of an 18-segment model [20]. Mechanical dispersion was quantified as the standard deviation of the time-to-peak negative longitudinal strain across the 12 basal and midventricular segments.

Measurement of resting blood pressure in combination with valvular event timing was used by an automated software to create a systolic pressure curve. Valvular event timing was derived from three apical views to mark moments in the cardiac cycle related to valve motion. By multiplying the results of the systolic pressure curve with strain rate measurements from longitudinal strain analysis, an LV power function was generated. Myocardial work was obtained by calculating the integral of the LV power function. Global constructive work (GCW) was derived by combining positive work during isovolumetric contraction and negative work during isovolumetric relaxation before mitral valve opening [21].

### Outcomes

Outcomes were predefined and included mean difference in changes from baseline to follow-up between the control group and high-normal potassium group in the systolic parameters: GLS, GCW, and LVEF, and the diastolic parameters e’ and E/e’.

### Statistical analysis

Continuous variables are reported as mean ± standard deviation or median with interquartile range (IQR) as appropriate. Categorical variables are reported as absolute numbers and relative frequencies. Continuous variables following a normal distribution were compared using Welch two-sample t-test. Within-individual comparisons between baseline and follow-up echocardiographic measurements were performed using the paired t-test. The Pearson chi-square test was used to compare categorical variables. The mean difference in changes was calculated as the between group difference in paired estimates. Normality was assessed visually using QQ-plots and homogeneity of variance was evaluated using residual plots. The mean difference in changes was compared between groups using Welch two-sample t-test. The presented results represent absolute change between study groups. Estimated differences are presented with 95% confidence intervals (CIs). Simple linear regression was used to investigate potential associations between p-K and different echocardiographic outcomes and correlations were assessed. Analysis of variance was used to compare the linear regression models of each echocardiographic parameter between the two groups. The odds ratio was determined for improvement in diastolic dysfunction grade between the two groups. Assumptions were tested prior to investigating data with t-test and linear regression models. Data analysis was performed according to the intention-to-treat principle. A two-sided P-value of < 0.05 was considered statistically significant. All analyses were performed using R ver. 4.4.1 (R Foundation for Statistical Computing).

### Sample size calculation

A mean difference in change in GLS of 1% was detected in our previous study [16]. In the current study, we assumed a smaller effect size of 0.5% with a higher standard deviation of 2.2, a two-tailed α = 0.05, and a power of 80% (1–β = 0.8) in a 1:1 randomization design. The values were chosen based on the results from our previous study and key differences in study design, including a longer follow-up period and a broader patient population in the current study. This calculation led to a sample size estimate of 146 patients per group. To account for potential dropouts, we rounded this up to 150 patients per group.

## Results

### Baseline

**Fig. 1:**
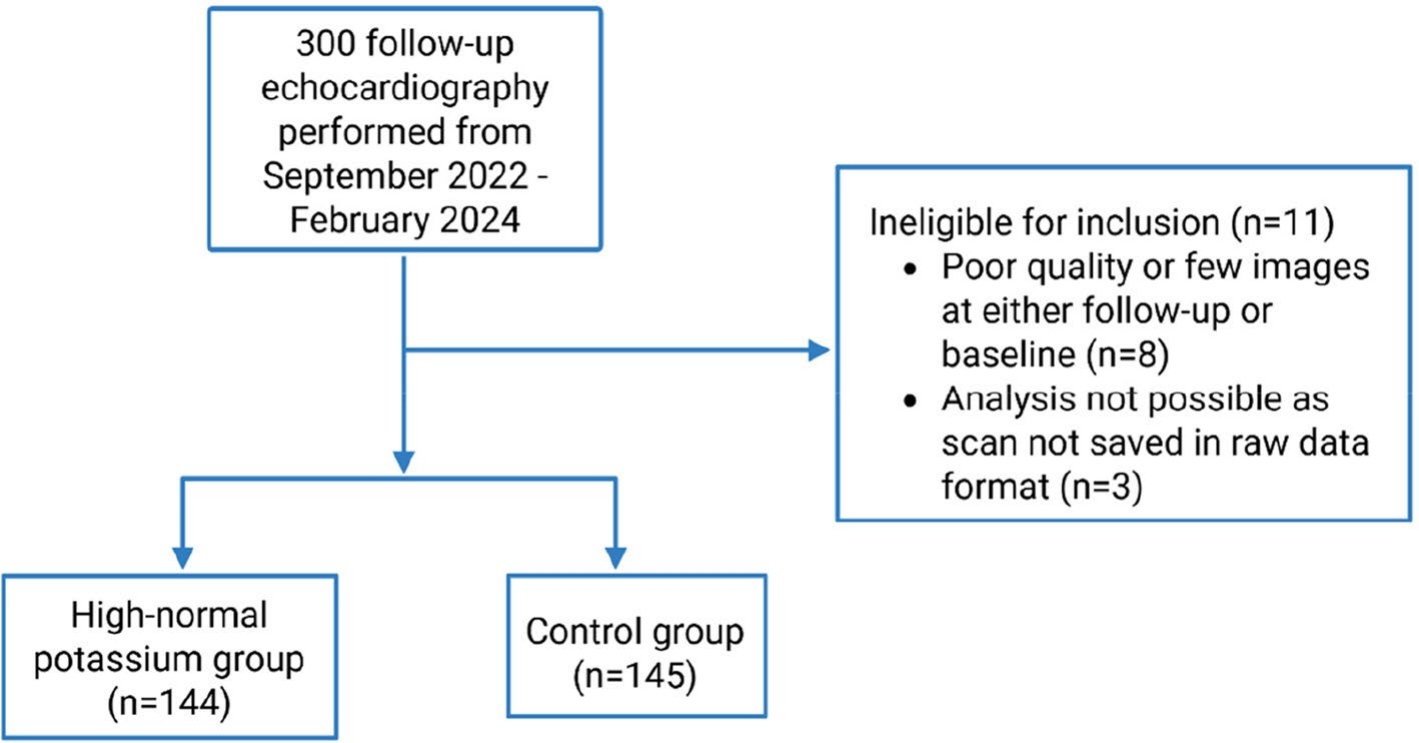
Flowchart of the study population. A total of 300 consecutive patients from the POCAST population were included. After exclusion due to poor image quality or missing data, the final study population comprised 289 patients (high-normal potassium group, n = 144; control group, n = 145)

A total of 300 follow-up echocardiography was performed and 289 patients (mean age, 58 ± 13.4 years; 77.5% male) were included. A flowchart of the enrollment process is presented in Fig. 1**.** The cohort consisted of 144 patients in the high-normal potassium group and 145 in the control group (Table 1). Among the included patients, a total of 121 (41.9%) had an ICD implanted for primary prevention and 168 (58.1%) for secondary prevention. Of these, 66 patients (22.8%) received a cardiac resynchronization therapy defibrillator (CRTD). For all but 18 patients, CRTD implantation occurred more than 1 year prior to study inclusion; these 18 patients were evenly distributed between the high-normal potassium group and control group. Ninety-eight patients (33.9%) had ischemic heart disease and 69 (23.9%) had a history of heart failure with reduced EF (HFrEF). There were no statistical differences in baseline parameters between the two groups, including comorbidities, blood pressure, p-K levels, kidney function and medications, except for a clinically insignificant lower body mass index in the high-normal potassium group (Table 1).

**Table 1:** Baseline characteristics of patients in the high-normal potassium and control groups (n = 289)

Characteristic	Control group (n = 145)	High-normal potassium group (n = 144)	P-value^a^
Male sex	115 (79.3)	109 (75.7)	0.462
Age (yr)	58.5 ± 13.0	57.7 ± 13.8	0.643
HFrEF (LVEF < 40%)	40 (27.6)	29 (20.1)	0.189
HFmrEF (LVEF, 40%–49%)	42 (29.0)	40 (27.8)	0.832
HFpEF (LVEF > 50% or normal)	63 (43.4)	74 (51.4)	0.353
Body mass index (kg/m^2^)	28.1 ± 5.0	26.4 ± 4.1	0.002
Previous AMI	33 (22.8)	35 (24.3)	0.757
Ischemic heart disease	51 (35.2)	47 (32.6)	0.649
Atrial fibrillation	50 (34.5)	41 (28.5)	0.271
Heart failure	66 (45.5)	68 (47.2)	0.771
Hypertension	69 (47.6)	62 (43.1)	0.439
Diabetes	17 (11.7)	11 (7.6)	0.240
ICD indication			0.433
Primary	64 (44.1)	57 (39.6)	
Secondary	81 (55.9)	87 (60.4)	
CRTD	33 (22.8)	33 (22.9)	0.913
Systolic blood pressure (mmHg)	129.0 ± 19.0	127.7 ± 18.9	0.596
Diastolic blood pressure (mmHg)	80.2 ± 11.4	79.5 ± 11.9	0.619
p-K (mmol/L)	4.0 ± 0.2	4.0 ± 0.2	0.389
p-Na (mmol/L)	140.3 ± 2.4	140.1 ± 2.5	0.501
eGFR (mL/min/1.73 m^2^)	78.2 ± 13.5	79.3 ± 12.8	0.530
Potassium supplement	28 (19.3)	29 (20.1)	0.882
Mineralocorticoid antagonist	36 (24.8)	45 (31.2)	0.237
β-Blocker	110 (75.9)	108 (75,0)	0.781
ACE inhibitor/angiotensin II receptor blocker	86 (59.3)	89 (61.8)	0.829
SGLT2 inhibitor	12 (8.3)	9 (6.2)	0.512
Entresto	14 (9.7)	17 (11.8)	0.555

Values are presented as number (%) or mean ± standard deviation

ACE, angiotensin-converting enzyme; AMI, acute myocardial infarction; CRTD, cardiac resynchronization therapy defibrillator; eGFR, estimated glomerular filtration rate; HFmrEF, heart failure with mildly reduced ejection fraction; HFpEF, heart failure with preserved ejection fraction; HFrEF, heart failure with reduced ejection fraction; ICD, implantable cardioverter-defibrillator; LVEF, left ventricular ejection fraction; p-K, plasma potassium; p-Na, plasma sodium; SGLT2, sodium-glucose cotransporter 2

^a^Pearson chi-square test was used for categorical variable and Welch two-sample t-test for continuous variables

For predefined echocardiographic parameters, we found the following mean baseline values: GLS, –12.75% ± 4.41%; GCW, 1,728 ± 540 mmHg; LVEF, 47.3% ± 9.8%; e’, 6.7 ± 2.4 cm/sec; and E/e’, 9.35 ± 4.11. The mean baseline p-K was 4.0 ± 0.2 mmol/L. Baseline characteristics of patients in the current substudy were compared to those in the rest of the POTCAST trial. Patients in the substudy were significantly younger, had fewer cardiovascular-related diseases, better kidney function, and were less likely to receive potassium supplements or MRA (Table S1).

### Follow-up

Patients were followed for a median of 729 days (IQR, 511–986 days) between baseline and follow-up echocardiography. At follow-up, the mean p-K was 4.3 ± 0.4 mmol/L in the high-normal potassium group and 4.1 ± 0.3 mmol/L in the control group. The mean difference in changes in p-K between the two groups was 0.23 mmol/L (95% CI, 0.14–0.32; P < 0.001), with higher values observed in the high-normal potassium group. No changes in blood pressure were observed between groups.

### Medication and compliance

In the high-normal potassium group (n = 144) at follow-up, 44 patients (30.6%) received potassium supplements alone, 21 patients (14.6%) received MRA alone, and 70 patients (48.6%) received both. A total of nine patients (6.2%) in the high-normal potassium group were nonadherent to study medication. At baseline and follow-up, the mean daily dose of potassium supplements in the high-normal potassium group (n = 144) was 277.5 mg (approximately 4 mmol) and 2,661 mg (approximately 35 mmol), respectively. The mean treatment dose of MRA in the high-normal potassium group was 8.9 mg at baseline and 27.9 mg at follow-up. Usual therapy in control patients included treatment with potassium supplements alone in 28 patients, MRA treatment in 31 patients and both in 11 patients. The mean dose of potassium supplements in the control group (n = 145) was 392 mg (approximately 5 mmol) and the mean MRA dose at follow-up was 8.8 mg.

### Echocardiographic findings

#### Changes between groups from baseline to follow-up

**Fig. 2:**
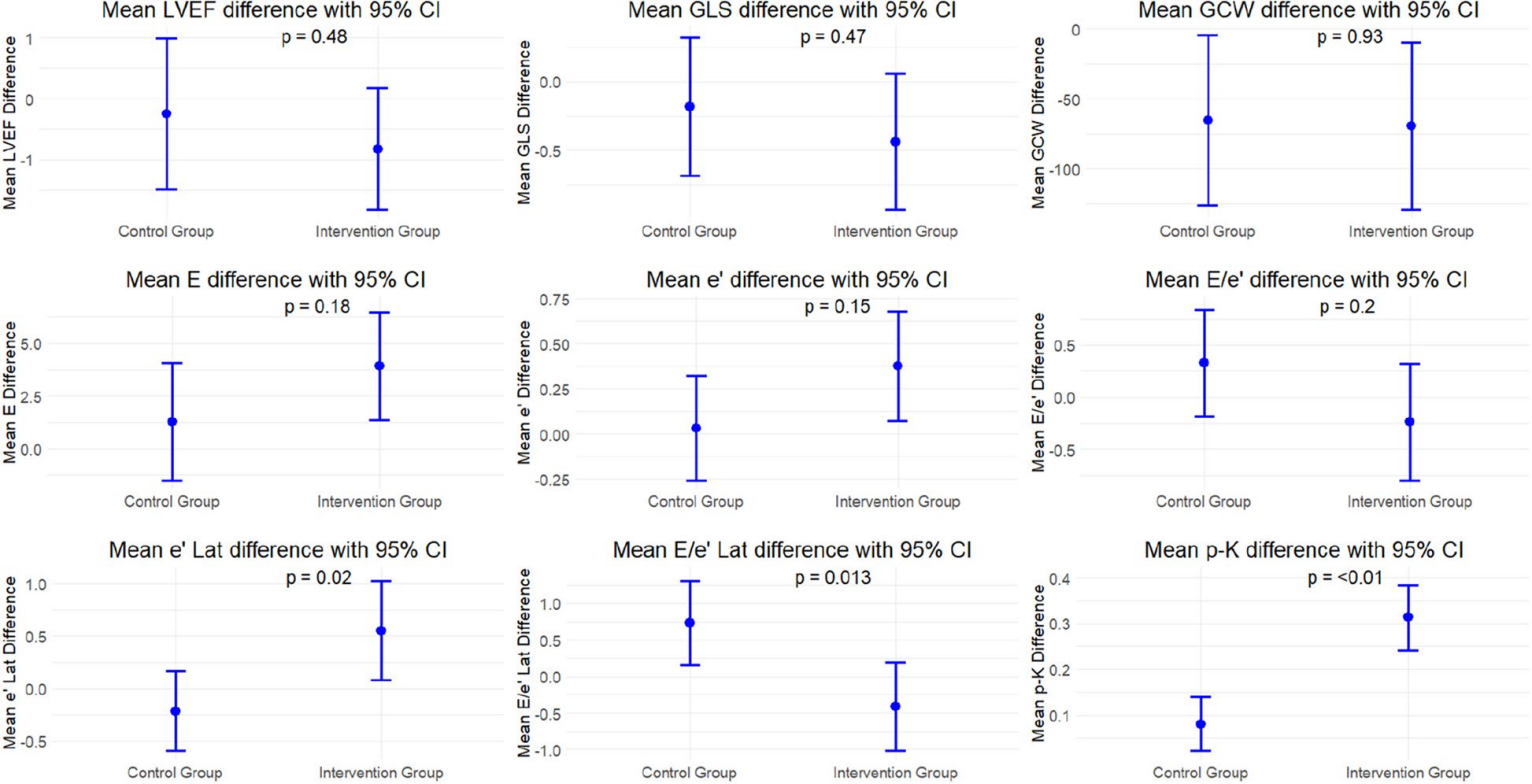
Error bar plots showing mean change from baseline with 95% confidence interval (CI) for eight echocardiographic variables and plasma potassium (p-K) in the control and high-normal potassium groups. GLS, global longitudinal strain; GCW, global constructive work; LVEF, left ventricular ejection fraction

No significant differences in systolic function changes, as assessed by the parameters GLS, GCW, and LVEF, were observed between the high-normal potassium and control group (Table 2). Similarly, no significant differences were found in diastolic parameters e’ and E/e’. However, the high-normal potassium group showed improvement in diastolic parameters e’lat and E/e’lat with a mean change difference of 0.77 cm/sec (95% CI, 0.12 to 1.40; P = 0.020) and –1.15 (95% CI, –2.1 to –0.25; P = 0.013), respectively. Figure 2 shows the different parameters using error bar plots. A subanalysis including only patients adherent to study medication in the high-normal potassium group, compared with control patients, showed similarly significant improvements in the high-normal potassium group for e’lat (mean difference in change, 0.77 cm/sec; 95% CI, 0.11 to 1.4; P = 0.023) and E/e’lat (mean difference in change, –1.12; 95% CI, –2.1 to –0.2; P = 0.019) (Table S2).

**Table 2:** Baseline and follow-up measurements and mean differences in clinical and echocardiographic parameters in the control and high-normal potassium groups

Parameter	Control group (n = 145)			High-normal potassium group (n = 144)			Mean difference in changes from baseline	P-value^b^
	Baseline	Follow-up	Mean difference^a^ (95% CI)	Baseline	Follow-up	Mean difference^a^ (95% CI)		
LVEF (%)	46.9 ± 9.6	46.6 ± 9.6	–0.24 (–1.5 to 1.0)	47.9 ± 9.6	47.1 ± 9.5	–0.8 (–1.8 to 0.18)	–0.6 (–2.17 to 1.02)	0.475
GLS (%)	–13.4 ± 4.0	–13.0 ± 3.9	0.4 (0.003 to 0.8)	–13.0 ± 3.9	–13.4 ± 4.0	0.2 (–0.2 to 0.64)	–0.26 (–0.8 to 0.4)	0.472
GCW (mmHg)	1,700.8 ± 533.2	1,635.4 ± 535.2	–65.4 (–127 to –4.3)	1,751.1 ± 539.7	1,681.7 ± 506.0	–69.4 (–130 to –9)	–3.99 (–89.5 to 81.6)	0.927
GWW (mmHg)	241.6 ± 147.6	257.3 ± 143.7	15.6 (–9.6 to 40.8)	248.1 ± 153.4	247.9 ± 137.7	–0.2 (–25.6 to 25.2)	–15.9 (–51.5 to 19.7)	0.381
MD (msec)	48.0 ± 5.7	47.2 ± 6.0	–0.8 (–1.9 to 0.24)	47.8 ± 5.6	47.0 ± 6.2	–0.7 (–1.7 to 0.18)	0.06 (–1.33 to 1.5)	0.912
E (cm/sec)	57.9 ± 18.2	59.2 ± 21.3	1.29 (–1.6 to 4.2)	55.4 ± 19.3	59.4 ± 21.8	3.9 (1.3 to 6.6)	2.6 (–1.3 to 6.6)	0.185
A (cm/sec)	52.6 ± 17.2	52.4 ± 17.3	–0.25 (–3.1 to 2.6)	53.0 ± 17.3	50.3 ± 17.8	–2.7 (–5.3 to –0.14)	–2.45 (–6.29 to 1.38)	0.209
E/A	1.1 ± 0.4	1.2 ± 0.5	0.04 (–0.04 to 0.12)	1.1 ± 0.5	1.2 ± 0.6	0.13 (0.06 to 0.21)	0.1 (–0.01 to 0.21)	0.080
e'sept (cm/sec)	5.8 ± 2.0	6.1 ± 2.3	0.3 (–0.06 to 0.57)	5.5 ± 2.0	5.9 ± 2.1	0.4 (0.08 to 0.64)	0.1 (–0.32 to 0.5)	0.627
e'lat (cm/sec)	8.0 ± 3.1	7.8 ± 3.3	–0.2 (–0.63 to 0.19)	7.7 ± 3.1	8.2 ± 3.3	0.6 (0.05 to 1.1)	0.77 (0.12 to 1.4)	0.020
e' (cm/sec)	6.9 ± 2.4	7.0 ± 2.5	0.034 (–0.3 to 0.36)	6.7 ± 2.2	7.1 ± 2.4	0.4 (0.04 to 0.71)	0.34 (–0.12 to 0.8)	0.149
E/e'sept	10.9 ± 4.6	10.7 ± 4.7	–0.2 (–1.0 to 0.48)	11.4 ± 6.2	11.0 ± 5.8	–0.3 (–1.1 to 0.49)	–0.1 (–1.17 to 0.96)	0.847
E/e'lat	8.2 ± 4.0	9.0 ± 4.6	0.7 (0.10 to 1.4)	8.5 ± 4.6	8.1 ± 4.7	–0.4 (–1.1 to 0.23)	–1.15 (–2.1 to –0.25)	0.013
E/e'	9.0 ± 3.6	9.3 ± 3.9	0.3 (–0.28 to 0.94)	9.1 ± 4.2	8.9 ± 3.8	–0.2 (–0.87 to 0.39)	–0.57 (–1.4 to 0.3)	0.198
LAVI (mL/m^2^)	34.9 ± 21.7	35.5 ± 18.3	0.6 (–1.3 to 2.6)	32.6 ± 15.2	33.8 ± 17.9	1.2 (–0.72 to 3.1)	0.55 (–2.15 to 3.26)	0.717
TR-gradient (mmHg)	23.6 ± 5.6	25.7 ± 7.3	2.1 (0.63 to 3.7)	23.8 ± 6.7	26.5 ± 9.3	2.7 (0.89 to 4.5)	0.54 (–1.8 to 2.9)	0.647
SBP (mmHg)	128.8 ± 17.5	122.6 ± 15.3	–6.1 (–9.1 to –3.1)	128.0 ± 20.3	121.3 ± 16.3	–6.7 (–9.3 to –4.1)	–0.59 (–4.5 to 3.4)	0.768
DBP (mmHg)	80.0 ± 11.2	74.8 ± 9.6	–5.3 (–7.2 to –3.3)	79.8 ± 12.1	75.4 ± 10.1	–4.4 (–6.2 to –2.6)	0.88 (–1.76 to 3.5)	0.513
p-K (mmol/L)	4.0 ± 0.2	4.1 ± 0.3	0.08 (0.02 to 0.14)	4.0 ± 0.2	4.3 ± 0.4	0.3 (0.23 to 0.37)	0.23 (0.14 to 0.32)	< 0.001

Values are presented as mean ± standard deviation, unless otherwise indicated

DBP, diastolic blood pressure; GCW, global constructive work; GLS, global longitudinal strain; GWW, global wasted work; LAVI, left atrial volume index; LVEF, left ventricular ejection fraction; MD, mechanical dispersion; p-K, plasma potassium; SBP, systolic blood pressure; TR, tricuspid regurgitation

^a^Difference between follow-up and baseline. ^b^Welch two-sample t-test used for continuous variables

Subgroup analysis of patients with HFrEF showed no significant differences between groups in changes across any of the prespecified outcomes (Table S3). Additionally, no significant difference in change in p-K was observed in patients with HFrEF between study groups (mean difference in change, 0.15; 95% CI, –0.05 to 0.35; P = 0.186).

Another subanalysis was performed in patients with LVEF 35% to 55%, consistent with the inclusion criteria of the previous POTCAST echocardiographic substudy [16]. No significant differences were observed in e’, E/e’, or GLS. However, a significant difference was observed in E/e’lat (mean difference in change, –1.08; 95% CI, –2.10 to –0.05; P = 0.040) (Table S4).

A final subanalysis excluding patients with CRTD showed results consistent with the main analysis, with no significant differences between groups in systolic or the prespecified diastolic parameters. However, the difference in E/e’lat remained significant (mean difference in change, –1.04; 95% CI, –2.10 to –0.06; P = 0.038) (Table S5).

#### Changes within the high-normal potassium group from baseline to follow-up

Changes in echocardiographic parameters within the high-normal potassium group are shown in Table 3. Significant improvements were observed in diastolic parameters, including E, E/A, tricuspid regurgitation gradient, e’ as well as e’sept and e’lat (all P < 0.05). Systolic parameters such as GLS and LVEF remained unchanged while GCW increased significantly (P = 0.018).

**Table 3:** Paired echocardiographic data at baseline and follow-up in the high-normal potassium group (n = 144)

Parameter	Baseline	Follow–up	Mean difference (95% CI)	P-value^a^
LVEF (%)	47.9 ± 9.6	47.1 ± 9.5	–0.82 (–1.83 to 0.18)	0.108
GLS (%)	–13.01 ± 4.3	–13.5 ± 4.4	–0.44 (–0.94 to 0.06)	0.088
MD (msec)	47.8 ± 5.6	47.0 ± 6.2	–0.75 (–1.68 to 0.18)	0.110
GCW (mmHg)	1,751.1 ± 539.7	1,681.7 ± 506.1	–69.4 (–129.8 to –9)	0.018
E (cm/sec)	55.5 ± 19.4	59.4 ± 21.8	3.86 (1.2 to 6.5)	0.005
A (cm/sec)	53.0 ± 17.3	50.3 ± 17.8	–2.70 (–5.26 to –0.14)	0.036
E/A	1.1 ± 0.5	1.2 ± 0.6	0.13 (0.06 to 0.21)	0.001
e'sept (cm/sec)	5.5 ± 2.0	5.9 ± 2.1	0.37 (0.09 to 0.64)	0.016
e'lat (cm/sec)	7.6 ± 3.1	8.2 ± 3.3	0.55 (0.06 to 1.05)	0.039
e' (cm/sec)	6.6 ± 2.3	7.0 ± 2.5	0.38 (0.05 to 0.72)	0.038
E/e’sept	11.5 ± 6.3	11.2 ± 6.0	–0.32 (–1.17 to 0.53)	0.464
E/e'lat	8.5 ± 4.7	8.1 ± 4.7	–0.42 (–1.07 to 0.22)	0.216
E/e'	9.3 ± 4.4	9.1 ± 4.3	–0.2 (–0.85 to 0.45)	0.553
LAVI (mL/m^2^)	32.4 ± 15.4	35.3 ± 26.1	1.3 (–0.64 to 3.3)	0.143
TR-gradient (mmHg)	23.9 ± 6.8	26.5 ± 9.3	2.67(0.85 to 4.49)	0.011

Values are presented as mean ± standard deviation, unless otherwise indicated

CI, confidence interval; GCW, global constructive work; GLS, global longi¬tudinal strain; LAVI, left atrial volume index; LVEF, left ventricular ejection fraction; MD, mechanical dispersion; TR, tricuspid regurgitation

^a^Paired t-test

### Diastolic dysfunction grading

**Fig. 3:**
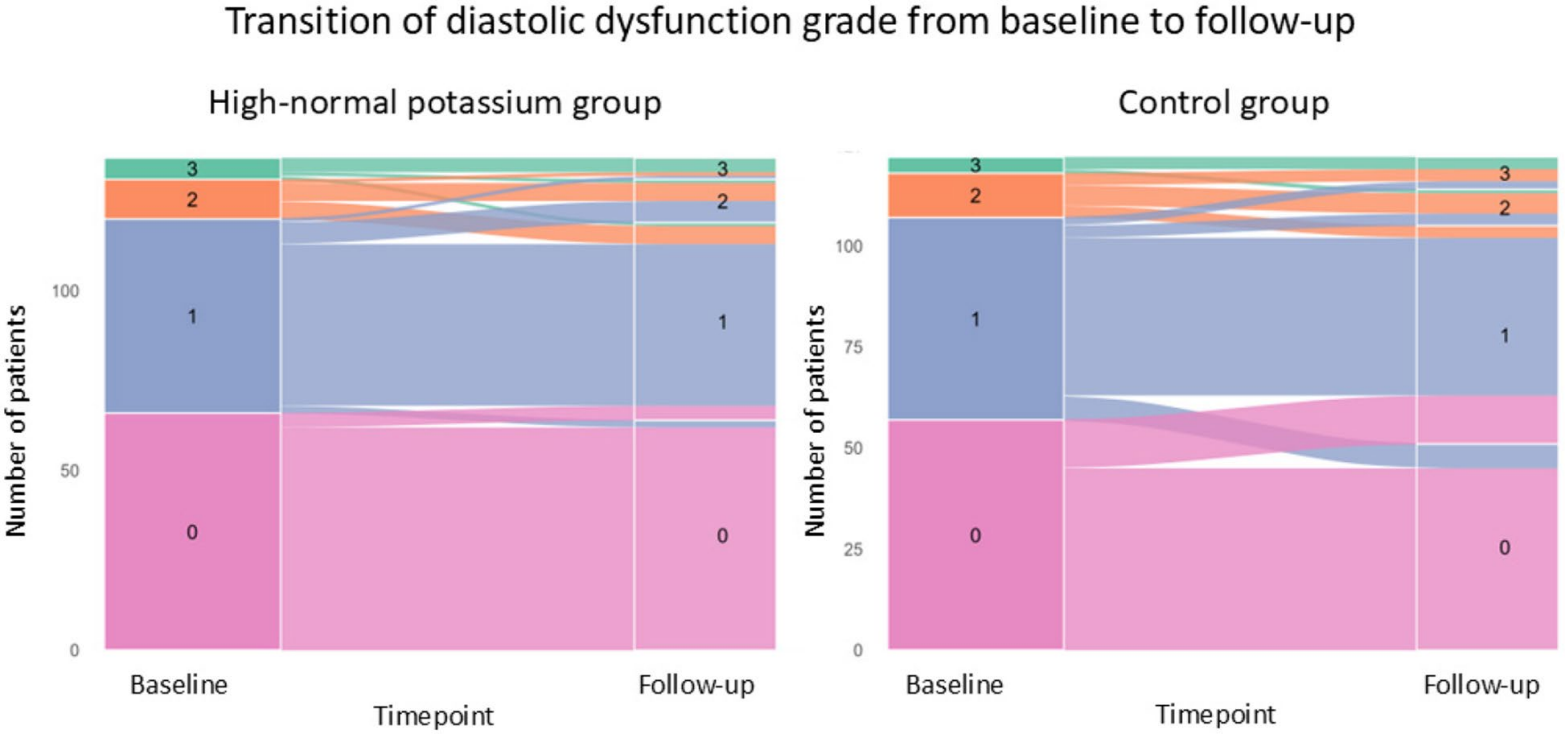
Alluvial plots showing transitions in diastolic dysfunction grade from baseline to follow-up in the high-normal potassium group and the control group

The odds ratio for improvement in diastolic dysfunction grade was 0.79 (95% CI, 0.30–2.02; P = 0.593) in the high-normal potassium group compared to the control group. A visualization of diastolic dysfunction grade at baseline and at follow-up in the control and high-normal potassium group is illustrated on Fig. 3.

### Relation between p-K and myocardial function

A simple linear regression model showed no significant associations between follow-up p-K levels and mean difference values in echocardiographic outcome variables regardless of the high-normal potassium groups. GLS (β = –0.17, P = 0.823, R^2^ = –0.003), LVEF (β = –0.90, P = 0.571, R^2^ = –0.002), GCW (β = –24.66, P = 0.767, R^2^ = –0.003), e’ (β = –0.04, P = 0.928, R^2^ = –0.004) or E/e’ (β = 0.23, P = 0.761, R^2^ = –0.004). Similarly, no associations were found between mean differences in p-K from baseline to follow-up or in mean differences of different echocardiographic parameters. The full panel of echocardiographic parameters can be found in Table 2.

## Discussion

This is the first clinical study to investigate the impact of potassium-increasing treatment on myocardial function after an extended period of treatment aiming at p-K in the high-normal range. Previous experimental and clinical studies have demonstrated acute or short-term associations between mechanical myocardial function and p-K values [13, 16, 22].

In this substudy of the POTCAST trial, we observed a change in the diastolic parameters e’lat (0.77 cm/sec) and E/e’lat (–1.15) that suggested a small improvement in diastolic function in patients receiving potassium-increasing treatment. These improvements persisted in high-normal potassium group patients who adhered to the study medication. However, no significant differences were observed in the septal or average values of these diastolic parameters.

These improvements, although modest, suggest a potential benefit of potassium-increasing treatment in enhancing diastolic function. No significant differences were found in systolic parameters LVEF, GLS, or GCW between study groups. Similarly, there was no difference in the change in diastolic dysfunction grade between groups.

Although diastolic dysfunction and indices such as E/e′ are established predictors of future cardiovascular events and all-cause mortality [23, 24], the magnitude of change observed in the present study was small and limited to lateral measurements rather than averaged indices. In the previous POTCAST echocardiographic substudy, larger changes were observed (e′ increase: 0.9 cm/sec [95% CI, 0.02 to 1.70], P = 0.04; E/e′ decrease: 1.5 [95% CI, − 2.9 to − 0.14], P = 0.03) [16]. Similarly, in a randomized trial of mineralocorticoid receptor antagonist therapy, e′ increased by 0.4 cm/sec (95% CI, 0.1 to 0.6; P = 0.002) and E/e′ decreased by 1.5 (95% CI, − 2.0 to − 0.9; P < 0.001) [25]. The minimal clinically important differences for these parameters are not well defined, but the effect sizes observed in the present study are small relative to changes reported in studies associated with clinical outcome benefits. Therefore, the observed improvements likely represent a modest physiological effect rather than a clearly clinically meaningful change at the patient level.

Finally, we found no associations between follow-up p-K levels or mean differences in p-K levels and any changes from baseline to follow-up of the predefined echocardiographic parameters, regardless of the high-normal potassium group.

### p-K and myocardial function

The association between p-K and myocardial function has been sparsely investigated. Experimental studies in both humans and dogs demonstrated that depletion of p-K led to impaired myocardial function [13, 22]. Fitzovich et al. [13] examined the effect of potassium depletion (p-K, 3.2 mmol/L) in dogs following epinephrine injection. Hypokalemic dogs exhibited a 33% lower relaxation response and a 60% higher peak rate of change in ejection power compared to normokalaemic dogs. Similarly, a human study compared the effects of 7 days of potassium-lowering treatment (p-K, 3.5 mmol/L) and potassium-increasing treatment (p-K, 4.6 mmol/L) and found that potassium depletion was associated with impaired diastolic myocardial function [22]. In addition, both studies showed significant variations in p-K levels between the normal and depleted states, suggesting that a substantial change in p-K might be required to detect a measurable effect on myocardial function.

Potassium homeostasis is closely regulated by various hormonal mechanisms, including the renin–angiotensin–aldosterone system. The mineralocorticoid aldosterone increases renal potassium excretion, leading to a decrease in p-K levels [26]. A study in patients with primary aldosteronism showed an association between higher aldosterone levels and lower GLS [27]. Furthermore, GLS findings were significantly associated with the serum potassium levels of the patients. While all of the above-mentioned studies suggested that myocardial function might be affected by changes in p-K, none of the studies evaluated whether these changes persist after a longer period of time.

The effects of long-term (6–15 months) MRA treatment on diastolic function in patients with heart failure with preserved EF has been evaluated in a meta-analysis. Eleven randomized controlled trials were included in the analysis, which revealed improvements in the diastolic parameter E/e’, a decrease in a surrogate marker for myocardial fibrosis, and a significant increase in serum potassium levels in patients receiving MRA treatment [28]. As elevated levels of aldosterone promote cardiac hypertrophy and fibrosis, the beneficial effects of MRA might be exclusively due to inhibition of aldosterone versus direct potassium modulation [29]. However, the impacts of MRA-induced increases in p-K on myocardial mechanical function remain unclear. In our study we observed a significant difference in p-K change between the groups along with differences in diastolic parameters, suggesting that potassium increase caused by MRA might still have an effect that is not fully understood.

We previously evaluated myocardial function in relation to potassium-increasing treatment in 50 patients over 6 weeks [16]. The study showed improvements in both diastolic parameters (average e’ and E/e’) and a systolic parameter (GLS) in patients receiving potassium supplements. Notably, the high-normal potassium group had a mean difference in change in p-K of 0.52 mmol/L compared to the control group. In the current substudy, we observed results consistent with previous findings regarding diastolic function and changes in p-K, although to a lesser degree. However, unlike the previous substudy, we found no associations with systolic parameters. Several differences between the two studies may explain these findings. First, while the previous randomized substudy included only patients with an LVEF of 35% to 55%, the current substudy aimed to represent a broader unselected ICD cohort from a contemporary Danish ICD population. Second, the mean difference in p-K changes in the current study (0.23 mmol/L) was lower than in the previous study (0.52 mmol/L). This could be attributed to a greater level of nonadherence to study medication over the longer follow-up period. Smaller p-K changes might result in subtle myocardial changes that are not detectable on echocardiography. Additionally, longer-term potassium-increasing treatment may lead to physiological adaptations or intracellular potassium redistribution, which could attenuate measurable systolic responses compared with short-term exposure.

Prolonged potassium-increasing treatment may lead to p-K stabilization, which could also explain the lower change in mean p-K difference in the current study. A previous POTCAST study using whole-body-counter measurement found that p-K was significantly elevated at 6 weeks but not at 6 months, whereas total body potassium was significantly elevated at 6 months but not at 6 weeks [30]. This suggests that potassium may shift intracellularly over time, raising the question of whether p-K is the best marker for assessing the long-term effects of potassium therapy. Furthermore, the gradual intracellular redistribution of potassium may establish a new steady state, potentially diminishing the beneficial mechanical effects observed in short-term treatment.

Given the substantial differences between the two studies, our findings provide novel insights into the long-term effects of potassium-increasing treatment, suggesting that it does not seem to consistently enhance myocardial function over extended periods.

### Plasma potassium and clinical outcomes

Studies on MRA use in patients with heart failure have demonstrated clinical improvements, potentially driven by subtle enhancements in mechanical function of the myocardium [11, 12]. These findings include increased survival rates as well and a reduced risk of hospitalization for worsening heart failure. These studies also reported significantly increased p-K levels in MRA-treated patients compared with controls. Many MRA studies are conducted in patients with reduced EF, suggesting better outcomes in these patients.

While the effect size of potassium intervention on the parameters e’lat and E/e’lat demonstrated in this study was small, we did not assess potential protective effects on cardiovascular outcomes. These outcomes will be evaluated when the POTCAST trial has ended. However, our findings suggest that any potential protective effect on arrhythmia observed in the POTCAST trial is unlikely to be mediated by improved myocardial function in response to p-K changes.

### Limitations

A small selection bias was identified when comparing the baseline characteristics of the study cohort and remaining patients in the POTCAST trial; however, this study’s population was still heterogenous in both myocardial function and medical history, as would be expected from an ICD cohort. Potassium-increasing treatment may have different effects across patient subgroups, particularly with respect to myocardial function. Therefore, studies involving more homogenous patient cohorts in terms of myocardial function may reveal more pronounced effects of p-K-increasing treatment. In the present study, subgroup analyses were conducted to explore the potential effects of p-K-increasing treatment in patients with HFrEF and in those with an LVEF of 35% to 55%. No statistically significant differences were observed, but the possible effects of limited statistical power cannot be excluded.

## Conclusions

Long-term potassium-increasing treatment leads to minor improvements in diastolic function but does not enhance systolic myocardial function on echocardiography. Additionally, no significant associations were found between p-K levels and changes in systolic and diastolic echocardiographic parameters over time.

## Supplementary Information

Additional file 1: Table S1. Comparison of baseline characteristics of patients from the current substudy and the re-maining patients from the POTCAST study. Table S2. Mean differences in clinical and echocardiographic parameters from baseline and follow-up among patients in the high-normal potassium group receiving study medication. Table S3. Mean differences in echocardiographic parameters between the control and high-normal potassium groups with HFrEF. Table S4. Mean differences in clinical and echocardiographic parameters from baseline to follow-up among patients with LVEF 35%-55%, consistent with the inclusion criteria of the first echocardiographic POTCAST substudy.

## Data Availability

Due to legal and ethical restrictions related to patient confidentiality and GDPR regulations, the data used in this study cannot be publicly shared.

## Abbreviations

CI: Confidence interval
CRTD: Cardiac resynchronization therapy defibrillator
EF: Ejection fraction
GCW: Global constructive work
GLS: Global longitudinal strain
HFrEF: Heart failure with reduced ejection fraction
ICD: Implantable cardioverter-defibrillator
IQR: Interquartile range
LV: Left ventricular
LVEF: Left ventricular ejection fraction
MRA: Mineralocorticoid receptor antagonist
p-K: Plasma potassium

## References

[1] Gumz ML, Rabinowitz L, Wingo CS. An integrated view of potassium homeostasis. N Engl J Med. 2015;373:60–72. 26132942 10.1056/NEJMra1313341 PMC5675534

[2] McDonough AA, Youn JH. Potassium homeostasis: the knowns, the unknowns, and the health benefits. Physiology (Bethesda). 2017;32:100–11. 28202621 10.1152/physiol.00022.2016 PMC5337831

[3] Ravens U, Cerbai E. Role of potassium currents in cardiac arrhythmias. Europace. 2008;10:1133–7. 18653669 10.1093/europace/eun193

[4] Hoss S, Elizur Y, Luria D, Keren A, Lotan C, Gotsman I, et al. Serum potassium levels and outcome in patients with chronic heart failure. Am J Cardiol. 2016;118:1868–74. 27726855 10.1016/j.amjcard.2016.08.078

[5] Macdonald JE, Struthers AD. What is the optimal serum potassium level in cardiovascular patients? J Am Coll Cardiol. 2004;43:155–61. 14736430 10.1016/j.jacc.2003.06.021

[6] Podrid PJ. Potassium and ventricular arrhythmias. Am J Cardiol. 1990;65:33E-44E. 2178376 10.1016/0002-9149(90)90250-5

[7] Weiss JN, Qu Z, Shivkumar K. Electrophysiology of hypokalemia and hyperkalemia. Circ Arrhythm Electrophysiol. 2017;10:e004667. 28314851 10.1161/CIRCEP.116.004667 PMC5399982

[8] Aldahl M, Jensen AC, Davidsen L, Eriksen MA, Møller Hansen S, Nielsen BJ, et al. Associations of serum potassium levels with mortality in chronic heart failure patients. Eur Heart J. 2017;38:2890–6. 29019614 10.1093/eurheartj/ehx460

[9] Wei X, Yohannan S, Richards JR. Physiology, cardiac repolarization dispersion and reserve. In: StatPearls. StatPearls Publishing; [updated 2023;. Available from: https://www.ncbi.nlm.nih.gov/books/NBK537194/. Cited 2026 jan 28. 30725879

[10] Pitt B, Remme W, Zannad F, Neaton J, Martinez F, Roniker B, et al. Eplerenone, a selective aldosterone blocker, in patients with left ventricular dysfunction after myocardial infarction. N Engl J Med. 2003;348:1309–21. 12668699 10.1056/NEJMoa030207

[11] Zannad F, McMurray JJ, Krum H, van Veldhuisen DJ, Swedberg K, Shi H, et al. Eplerenone in patients with systolic heart failure and mild symptoms. N Engl J Med. 2011;364:11–21. 21073363 10.1056/NEJMoa1009492

[12] Pitt B, Zannad F, Remme WJ, Cody R, Castaigne A, Perez A, et al. The effect of spironolactone on morbidity and mortality in patients with severe heart failure. N Engl J Med. 1999;341:709–17 (**Randomized Aldactone Evaluation Study Investigators**). 10471456 10.1056/NEJM199909023411001

[13] Fitzovich DE, Hamaguchi M, Tull WB, Young DB. Chronic hypokalemia and the left ventricular responses to epinephrine and preload. J Am Coll Cardiol. 1991;18:1105–11. 1894855 10.1016/0735-1097(91)90774-4

[14] Kurisu S, Iwasaki T, Mitsuba N, Ishibashi K, Dohi Y, Nishioka K, et al. Effects of serum potassium level on left ventricular diastolic function in patients with primary aldosteronism. Int J Cardiol. 2012;160:68–70. 22713679 10.1016/j.ijcard.2012.05.085

[15] Wang D, Xu JZ, Chen X, Chen Y, Shao S, Zhang W, et al. Speckle-tracking echocardiographic layer-specific strain analysis on subclinical left ventricular dysfunction in patients with primary aldosteronism. Am J Hypertens. 2019;32:155–62. 30462153 10.1093/ajh/hpy175

[16] Winsløw U, Sakthivel T, Zheng C, Philbert B, Vinther M, Frandsen E, et al. The effect of increased plasma potassium on myocardial function; a randomized POTCAST substudy. Int J Cardiovasc Imaging. 2023;39:2097–106. 37470856 10.1007/s10554-023-02914-x PMC10673982

[17] Winsløw U, Sakthivel T, Zheng C, Bosselmann H, Haugan K, Bruun N, et al. Targeted potassium levels to decrease arrhythmia burden in high risk patients with cardiovascular diseases (POTCAST): study protocol for a randomized controlled trial. Am Heart J. 2022;253:59–66. 35835265 10.1016/j.ahj.2022.07.003

[18] Jøns C, Zheng C, Winsløw UC, Danielsen EM, Sakthivel T, Frandsen EA, et al. Increasing the potassium level in patients at high risk for ventricular arrhythmias. N Engl J Med. 2025;393:1979–89. 40879429 10.1056/NEJMoa2509542

[19] Nagueh SF, Smiseth OA, Appleton CP, Byrd BF, Dokainish H, Edvardsen T, et al. Recommendations for the evaluation of left ventricular diastolic function by echocardiography: an update from the American Society of Echocardiography and the European Association of Cardiovascular Imaging. J Am Soc Echocardiogr. 2016;29:277–314. 27037982 10.1016/j.echo.2016.01.011

[20] Kalam K, Otahal P, Marwick TH. Prognostic implications of global LV dysfunction: a systematic review and meta-analysis of global longitudinal strain and ejection fraction. Heart. 2014;100:1673–80. 24860005 10.1136/heartjnl-2014-305538

[21] Saffi H, Winsløw U, Sakthivel T, Højgaard EV, Linde J, Philbert B, et al. Global constructive work is associated with ventricular arrhythmias after cardiac resynchronization therapy. Eur Heart J Cardiovasc Imaging. 2023;25:29–36. 37490039 10.1093/ehjci/jead180

[22] Srivastava TN, Young DB. Impairment of cardiac function by moderate potassium depletion. J Card Fail. 1995;1:195–200. 9420651 10.1016/1071-9164(95)90024-1

[23] Zhou D, Huang Y, Fu M, Cai A, Tang S, Feng Y. Prognostic value of tissue Doppler E/e’ ratio in hypertension patients with preserved left ventricular ejection fraction. Clin Exp Hypertens. 2018;40:554–9. 29400582 10.1080/10641963.2017.1407332

[24] Halley CM, Houghtaling PL, Khalil MK, Thomas JD, Jaber WA. Mortality rate in patients with diastolic dysfunction and normal systolic function. Arch Intern Med. 2011;171:1082–7. 21709107 10.1001/archinternmed.2011.244

[25] Edelmann F, Wachter R, Schmidt AG, Kraigher-Krainer E, Colantonio C, Kamke W, et al. Effect of spironolactone on diastolic function and exercise capacity in patients with heart failure with preserved ejection fraction: the Aldo-DHF randomized controlled trial. JAMA. 2013;309:781–91. 23443441 10.1001/jama.2013.905

[26] Palmer BF, Clegg DJ. Physiology and pathophysiology of potassium homeostasis. Adv Physiol Educ. 2016;40:480–90. 27756725 10.1152/advan.00121.2016

[27] Chen ZW, Huang KC, Lee JK, Lin LC, Chen CW, Chang YY, et al. Aldosterone induces left ventricular subclinical systolic dysfunction: a strain imaging study. J Hypertens. 2018;36:353–60. 28902663 10.1097/HJH.0000000000001534

[28] Pandey A, Garg S, Matulevicius SA, Shah AM, Garg J, Drazner MH, et al. Effect of mineralocorticoid receptor antagonists on cardiac structure and function in patients with diastolic dysfunction and heart failure with preserved ejection fraction: a meta-analysis and systematic review. J Am Heart Assoc. 2015;4:e002137. 26459931 10.1161/JAHA.115.002137 PMC4845109

[29] Ferrario CM, Schiffrin EL. Role of mineralocorticoid receptor antagonists in cardiovascular disease. Circ Res. 2015;116:206–13. 25552697 10.1161/CIRCRESAHA.116.302706 PMC4283558

[30] Winsløw U, Sakthivel T, Zheng C, Bosselmann H, Haugan K, Bruun N, et al. Treatment-induced increase in total body potassium in patients at high risk of ventricular arrhythmias; a randomized POTCAST substudy. PLoS ONE. 2023;18:e0288756. 37467227 10.1371/journal.pone.0288756 PMC10355384

