# Supplementary material for: Effects of plasma potassium on myocardial function: a POTCAST substudy
Source: J Cardiovasc Imaging. 2026 Apr 2;34:7. doi: 10.1186/s44348-026-00068-7 (PMC13045081; doi:10.1186/s44348-026-00068-7)
Supplement: Supplementary file 1 — Additional file 1: Table S1. Comparison of baseline characteristics of patients from the current substudy and the re-maining patients from the POTCAST study. Table S2. Mean differences in clinical and echocardiographic parameters from baseline and follow-up among patients in the high-normal potassium group receiving study medication. Table S3. Mean differences in echocardiographic parameters between the control and high-normal potassium groups with HFrEF. Table S4. Mean differences in clinical and echocardiographic parameters from baseline to follow-up among patients with LVEF 35%-55%, consistent with the inclusion criteria of the first echocardiographic POTCAST substudy. [file 44348_2026_68_MOESM1_ESM.docx]

**Supplementary Appendix**

Effect of plasma potassium on myocardial function: A POTCAST substudy

**Table S1.** Comparison of baseline characteristics of patients from the current substudy and the remaining patients from the POTCAST study

|  | POTCAST  N = 779^1^ | Substudy  N = 289^1^ | p-value^2^ |
| --- | --- | --- | --- |
| Male sex, n(%) | 615 (79%) | 224 (78%) | 0.621 |
| Age, years | 64.0 (11.2) | 58.1 (13.4) | <0.001 |
| BMI, kg/m^2^ | 28.5 (5.1) | 27.3 (4.6) | <0.001 |
| Previous AMI, n(%) | 303 (39%) | 68 (24%) | <0.001 |
| IHD, n(%) | 410 (53%) | 98 (34%) | <0.001 |
| Afib, n(%) | 253 (32%) | 91 (31%) | 0.833 |
| Heart Failure, n(%) | 454 (58%) | 134 (46%) | <0.001 |
| Hypertension, n(%) | 404 (52%) | 131 (45%) | 0.042 |
| Diabetes, n(%) | 151 (19%) | 28 (9.7%) | <0.001 |
| ICD Indication |  |  | 0.824 |
| Primary, n(%) | 335 (43%) | 122 (42%) |  |
| Secondary, n(%) | 443 (57%) | 167 (58%) |  |
| Systolic blood pressure, mmHg | 129.3 (19.4) | 128.5 (19.3) | 0.477 |
| Diastolic blood pressure, mmHg | 78.9 (11.4) | 79.7 (11.8) | 0.323 |
| Potassium Supplements, n(%) | 202 (27%) | 57 (20%) | 0.032 |
| Mineralocorticoid antagonists, n (%) | 314 (41%) | 81 (29%) | <0.001 |
| Beta Blockers, n(%) | 619 (80%) | 218 (76%) | 0.144 |
| ACE inhibitors or Angiotensin II Inhibitors, n(%) | 567 (74%) | 175 (61%) | <0.001 |
| p-K, mmol/l | 4.0 (0.2) | 4.0 (0.2) | 0.304 |
| p-Na, mmol/l | 139.8 (2.4) | 140.2 (2.5) | 0.013 |
| eGFR, ml/min/1.73 m^2^ | 76.8 (14.5) | 78.7 (13.3) | 0.038 |
| ¹ Values are presented as mean (±SD) or number (%). ² Welch’s t-test.  **Abbreviations:** A = late diastolic mitral inflow velocity; ACE = angiotensin-converting enzyme; AF = atrial fibrillation; AMI = acute myocardial infarction; BMI = body mass index; eGFR = estimated glomerular filtration rate; ICD = implantable cardioverter-defibrillator; IHD = ischemic heart disease; p-K = plasma potassium; p-Na = plasma sodium. | | | |

**Table S2.** Mean differences in clinical and echocardiographic parameters from baseline and follow-up among patients in the high-normal potassium group receiving study medication

|  | **Control**  N = 145^1^ | **High-normal potassium**  N = 135^1^ | **p-value**^2^ |
| --- | --- | --- | --- |
| LVEF, % | -0.2 (7.5) | -0.9 (6.2) | 0.463 |
| GLS, % | -0.2 (3.1) | -0.5 (3.0) | 0.444 |
| GCW, mmHg | -65.4 (370.9) | -78.4 (370.1) | 0.791 |
| e'sept, cm/s | 0.3 (1.7) | 0.3 (1.6) | 0.922 |
| e'lat, cm/s | -0.2 (2.3) | 0.6 (2.9) | 0.023 |
| e', cm/s | 0.0 (1.8) | 0.3 (1.9) | 0.197 |
| E/e'sept | -0.2 (3.7) | -0.2 (4.6) | 0.928 |
| E/e'lat | 0.7 (3.5) | -0.4 (3.8) | 0.019 |
| E/e' | 0.3 (3.1) | -0.2 (3.5) | 0.345 |
| p-K difference, mmol/L | 0.1 (0.4) | 0.3 (0.4) | <0.001 |
| ¹ Values are presented as mean (±SD) or number (%). ² Welch’s t-test.  **Abbreviations:** e′ = average of septal and lateral early diastolic mitral annular velocity; GCW = global constructive work; GLS = global longitudinal strain; LVEF = left ventricular ejection fraction; p-K = plasma potassium. | | | |

**Table S3.** Mean differences in echocardiographic parameters between the control and high-normal potassium groups with HFrEF

|  | **Control**   N = 41^1^ | **High-normal potassium**  N = 34^1^ | **p-value**^2^ |
| --- | --- | --- | --- |
| LVEF, % | 2.4 (8.0) | 2.1 (8.0) | 0.877 |
| GLS, % | -0.4 (2.9) | -1.6 (3.4) | 0.124 |
| GCW, mmHg | -27.1 (368.2) | 91.1 (396.5) | 0.221 |
| e'sept, cm/s | 0.3 (1.8) | 0.0 (2.0) | 0.691 |
| e'lat, cm/s | -0.6 (2.6) | -0.4 (3.0) | 0.841 |
| e', cm/s | -0.2 (2.1) | -0.3 (2.3) | 0.903 |
| E/e'sept | -0.7 (4.8) | -0.5 (7.6) | 0.932 |
| E/e'lat | 1.8 (5.2) | -0.2 (5.8) | 0.179 |
| E/e' | 0.9 (4.8) | -0.4 (6.2) | 0.443 |
| p-K difference, mmol/L | 0.1 (0.4) | 0.3 (0.5) | 0.186 |
| ¹ Values are presented as mean (±SD) or number (%). ² Welch’s t-test.  **Abbreviations:** e′ = average of septal and lateral early diastolic mitral annular velocity; GCW = global constructive work; GLS = global longitudinal strain; LVEF = left ventricular ejection fraction; p-K = plasma potassium. | | | |

**Table S4.** Mean differences in clinical and echocardiographic parameters from baseline to follow-up among patients with LVEF 35-55%, consistent with the inclusion criteria of the first echocardiographic POTCAST substudy.

|  | **Control**  N = 98^1^ | **High-normal potassium**  N = 92^1^ | **p-value**^2^ |
| --- | --- | --- | --- |
| LVEF, % | 0.9 (7.4) | -0.5 (5.9) | 0.143 |
| GLS, % | -0.4 (3.1) | -0.4 (2.8) | 0.962 |
| GCW, mmHg | -42.0 (380.2) | -62.4 (364.9) | 0.706 |
| e'lat, cm/s | 0.0 (2.2) | 0.7 (2.7) | 0.065 |
| e', cm/s | 0.1 (1.7) | 0.4 (1.6) | 0.321 |
| E/e'sept | 0.0 (3.9) | -0.3 (4.5) | 0.744 |
| E/e'lat | 0.7 (3.3) | -0.4 (3.4) | 0.040 |
| E/e' | 0.4 (3.0) | -0.1(3.0) | 0.352 |
| p-K difference, mmol/L | 0.1 (0.4) | 0.3 (0.5) | <0.001 |
| ¹ Values are presented as mean (±SD) or number (%). ² Welch’s t-test.  **Abbreviations:** e′ = average of septal and lateral early diastolic mitral annular velocity; GCW = global constructive work; GLS = global longitudinal strain; LVEF = left ventricular ejection fraction; p-K = plasma potassium | | | |
